# Cultivating competitive advantage for Chinese SMEs via big data analytics: The mediating role of data-driven innovation and antecedents of data governance and data-driven culture

**DOI:** 10.1371/journal.pone.0337324

**Published:** 2025-12-02

**Authors:** Song Rixin, Ramayah, Ma Xinrui

**Affiliations:** 1 School of Management, Universiti Sains Malaysia, Minden, Penang, Malaysia; 2 Sunway Business School (SBS), Sunway University, Petaling Jaya, Selangor, Malaysia; 3 Department of Information Technology & Management, Daffodil International University, Birulia, Bangladesh; 4 Faculty of Business, Sohar University, Sohar, Oman; 5 Asia Pacific University of Technology&Innovation (APU), Kuala Lumpur, Malaysia; 6 University Center for Research & Development (UCRD), Chandigarh University, Ludhiana, Punjab, India; 7 School of Business, The University of Jordan (UJ), Amman, Jordan; 8 Faculty of Economics and Business, Universitas Indonesia (UI), Depok City, West Java, Indonesia; 9 The College of Computing, Arts and Sciences, Lyceum of the Philippines University Batangas, Batangas City, Batangas, Philippines; University of Southampton, MALAYSIA

## Abstract

This study explores how small and medium-sized enterprises (SMEs) can leverage big data analytics (BDA) to gain competitive advantage (CA), highlighting the mediating role of data-driven innovation (DDI) and the foundational importance of data governance (DG) and data-driven culture (DDC). Despite the transformative potential of BDA, SMEs often struggle to translate data insights into competitive outcomes due to inadequate governance, cultural barriers, and a lack of clarity on how to operationalize analytics through innovation. Drawing on the resource-based view (RBV) and dynamic capability view (DCV), this study adopts a quantitative approach and analyzes survey data from 287 Chinese SMEs using partial least squares structural equation modeling (PLS-SEM). The results show that DG and DDC significantly enhance BDA, which directly improves CA and strongly drives DDI. DDI further mediates 61% of the total effect of BDA on CA, highlighting its key role in translating data insights into market innovation. The study advances the theoretical framework by positioning DG and DDC as valuable, scarce, inimitable and non-substitutable (VRIN) resources and DDI as a dynamic “access” mechanism. Practical insights urge SMEs to prioritize governance protocols, cultural alignment, and innovation channels to bridge the gap between analytical and competitive outcomes. Limitations include geographic focus and cross-sectional design, prompting future longitudinal and cross-industry investigations. This study provides a roadmap for SMEs to leverage data-driven strategies in turbulent markets.

## 1. Introduction

During an era of unparalleled digital transformation, small and medium-sized enterprises (SMEs)—which account for over 90% of enterprises and contribute more than 60% of GDP in China according to National Bureau of Statistics of China, 2024—navigate a business landscape of volatility, complexity, and intensified competition. The exponential advancement of big data technologies has radically reshaped organizational capabilities, enabling the collection and processing of large datasets with unparalleled precision [1]. Big data analytics (BDA)—defined as the use of advanced tools (e.g., machine learning, predictive modeling) to extract actionable insights from complex datasets [2]—has become a vital driver of competitive power for SMEs. Resource-constrained SMEs rely on BDA to optimize decision-making (e.g., demand forecasting for inventory management), predict market trends (e.g., customer preference shifts), and innovate strategically (e.g., tailoring products to niche needs) [3,4]. For instance, Chinese manufacturing SMEs using BDA for predictive maintenance reduced equipment downtime by 22% [5], while retail SMEs leveraging BDA for customer behavior analysis improved service customization and retention by 18% [6]. The value of BDA lies in its ability to turn fragmented data into actionable knowledge: it aids firms in understanding customer needs (e.g., identifying unmet demands via sentiment analysis), optimizing offerings (e.g., refining product features based on usage data), and formulating targeted market strategies (e.g., dynamic pricing models) [4,7]. Existing literature confirms that BDA radically enhances firms’ innovation capabilities—enabling faster R&D cycles and more targeted solutions—and helps derive lasting competitive strengths [4,8].

Data-driven innovation (DDI)—the development of new products, services, or processes based on BDA insights [9]—is the critical bridge between BDA and competitive advantage (CA). DDI positions data as both a foundation for decision-making and a source of new value: for example, a Chinese logistics SME used BDA-derived demand forecasting to launch a “dynamic route optimization” service, cutting transportation costs by 15% and gaining market share from larger rivals [10]. Through effective DDI, organizations can respond faster to market changes (e.g., adapting to new consumer trends) and boost innovation power—key to strengthening competitive advantage (CA)—a firm’s ability to outperform rivals via cost leadership, product differentiation, or service superiority [11]. For SMEs, DDI is particularly valuable: it allows them to compete with larger firms by focusing innovation on high-impact, data-backed opportunities rather than resource-intensive trial-and-error [12].

However, despite BDA’s and DDI’s significant potential, SMEs face substantial barriers to their practical application. These include limited capabilities in data integration and analysis (e.g., lack of skilled data analysts), insufficient understanding of DDI mechanisms (e.g., how to translate BDA insights into new products), and neglect of data governance (DG)—a framework of policies ensuring data quality, security, and compliance [13] and data-driven culture (DDC)—organizational norms of data-guided decision-making [14]—factors that underpin successful data-driven strategies [15–17]. For example, while studies have highlighted that firms with BDA technical skills can enhance business model innovation, there remains a critical gap in understanding how these skills are effectively translated into tangible business value—especially without DG and DDC [18]. Additionally, organizations often overlook DG’s role in mitigating risks (e.g., data silos, privacy breaches) and DDC’s role in fostering employee adoption of BDA, leading to inefficient use of analytical insights [15,19,20]. These challenges reveal three clear research gaps: (1) Most studies focus on BDA’s technical aspects (e.g., tools) while neglecting DG and DDC as key antecedents of BDA effectiveness in SMEs; (2) The mediating role of DDI between BDA and CA is underexamined, leaving a “black box” in how analytics translate to competitive outcomes; (3) Few studies integrate the Resource-Based View (RBV) and Dynamic Capabilities View (DCV) to explain how SMEs convert data resources into sustainable CA, resulting in fragmented theoretical understanding. These gaps hinder SMEs from fully leveraging BDA to achieve CA, creating a pressing need for a comprehensive framework that addresses both technical and organizational enablers of data-driven success.

Against this backdrop, this study aims to address three core objectives: (1) Identify the key antecedents of BDA effectiveness in SMEs, with a focus on data governance (DG) and data-driven culture (DDC); (2) Examine the direct and indirect effects of BDA on competitive advantage (CA), particularly through the mediating role of DDI; (3) Develop an integrated theoretical framework that bridges RBV and DCV to explain how SMEs can convert data resources into sustainable competitive advantage.

The contributions of this study are threefold: First, it fills a literature gap by systematically examining DG and DDC as critical enablers of BDA, addressing prior neglect of organizational factors in BDA research. Second, it clarifies the mechanism through which BDA drives CA by highlighting DDI as a key mediator, resolving ambiguity in how analytical capabilities translate to market outcomes. Third, it provides actionable insights for SMEs to prioritize governance, culture, and innovation channels, offering a practical roadmap for data-driven strategy implementation.

The remainder of this paper is structured as follows: Section 2 reviews relevant literature and develops hypotheses based on RBV and DCV. Section 3 describes the methodology, including sample selection, measurement instruments, and ethical considerations. Section 4 presents the analysis and results using PLS-SEM. Section 5 discusses the findings in relation to existing literature. Section 6 concludes with theoretical and managerial implications, limitations, and future research directions.

## 2. Literature review and hypotheses development

### 2.1. Big Data Analytics (BDA) in SMEs: Value, antecedents, and limitations

#### 2.1.1. Definition and strategic value of BDA for SMEs.

BDA is defined as the process of extracting actionable insights from large, heterogeneous datasets using advanced technologies (e.g., machine learning, predictive modeling, real-time analytics) to support decision-making, optimize operations, and identify market opportunities [2]. For SMEs, BDA serves as a “leveling tool” to compete with large firms by compensating for limitations in scale and capital.

Dubey, Gunasekaran [2] analyzed 320 global manufacturing SMEs and found that BDA reduces supply chain lead times by 20% through accurate demand forecasting and real-time supplier performance monitoring. Milić, Veljković [6] demonstrated that retail SMEs using BDA for customer behavior analysis improve service customization rates by 18%, enhancing customer retention. Zhou, Li [5] reported that Chinese manufacturing SMEs leveraging BDA for predictive maintenance cut equipment downtime by 22%, lowering operational costs by 15%.

#### 2.1.2. Overlooked antecedents: DG and DDC.

Despite consensus on BDA’s value, existing research disproportionately focuses on technological antecedents (e.g., software tools, data infrastructure) while neglecting organizational and cultural enablers—a critical oversight for SMEs.

Data governance (DG) defined as a “systematic framework of policies, processes, and responsibilities ensuring data quality, security, and regulatory compliance” (Khatri & Brown, 2010), DG is the foundation of reliable BDA. Pansara [21] surveyed 180 SMEs and found that 45% of BDA failures stem from poor DG (e.g., fragmented data ownership, unstandardized quality checks), leading to inaccurate analytical outputs. However, seminal BDA studies (e.g., Mikalef, Boura [1] and Dubey, Gunasekaran [2]) exclude DG from their frameworks, limiting their applicability to SMEs with fragile data management systems.

Data-driven culture (DDC) characterized by organizational norms that prioritize data-guided decision-making over intuition [14], DDC determines whether BDA insights are translated into action. Windt, Borgman [20] found that only 33% of SMEs have established a robust DDC, with employee resistance to data-driven practices reducing BDA adoption rates by 33%. Mikalef, Boura [1]—which explored BDA’s link to innovation in 251 firms—further exacerbates this gap by overrepresenting large enterprises (62% of the sample) and ignoring DDC, which is more influential in SMEs due to their flatter hierarchical structures.

### 2.2. Data-Driven Innovation (DDI) in SMEs: Role as a “translation mechanism”

#### 2.2.1. Definition and dimensions of DDI.

DDI refers to the development of new products, services, processes, or business models based on insights derived from BDA [9]. Unlike general innovation, DDI is distinguished by its data-centricity: data is not just a “supporting tool” but a “core input” for value creation [3]. For SMEs, DDI typically manifests in two dimensions: Incremental innovation: refinements to existing offerings (e.g., optimizing product features based on usage data). Radical innovation: breakthrough innovations (e.g., launching new business models via market gap analysis).

#### 2.2.2. Underexamined mediation: BDA→DDI→CA.

Studies on data-driven innovation (DDI) have emphasized its role in translating data into value [9] but failed to systematically examine its mediating effect between BDA and CA. Adi Sahputra and Nendi [3] conducted semi-structured interviews with 15 Indonesian SMEs and found that while BDA identifies customer pain points with 82% accuracy, only 35% of firms convert these insights into DDI. However, their qualitative design prevents quantifying DDI’s contribution to CA (e.g., market share growth, cost reduction). Al-Khatib [8] explored BDA→green DDI → CA in 189 SMEs in the MENA region’s environmental sector, finding that green radical DDI explains 38% of BDA’s impact on CA (Table 1). Yet the study’s focus on a single industry (green energy) limits generalizability to SMEs in manufacturing, retail, or logistics—sectors where DDI takes different forms (e.g., process optimization vs. product innovation).

**Table 1:** Comparative analysis of existing studies on BDA, DDI, and CA in SMEs.

Author(s) & Year	Research Focus	Methodology	Sample Characteristics	Key Findings	Limitations
Dubey, Gunasekaran [2]	BDA & supply chain performance in SMEs	Quantitative (PLS-SEM, n = 320)	Global manufacturing SMEs	BDA enhances operational efficiency via demand forecasting.	Neglects DG/DDC as BDA antecedents; no DDI/CA analysis.
Mikalef, Boura [1]	BDA, dynamic capabilities, & innovation	Quantitative (PLS-SEM, n = 251)	Mixed-size firms (62% large)	BDA drives innovation via sensing/seizing capabilities.	Overrepresents large firms; ignores DDC’s role in SMEs.
Al-Khatib [8]	BDA, green DDI, & CA	Quantitative (PLS-SEM, n = 189)	MENA region green industry SMEs	BDA improves CA through green radical innovation.	Limited to environmental sector; no DG measurement.
Sabir, Sohail [4]	BDA, data availability, & CA	Quantitative (PLS-SEM, n = 210)	Pakistani SMEs	Innovative capabilities mediate BDA-CA link.	Ignores DDI as a specific mediator; geographically limited.
Adi Sahputra and Nendi [3]	BDA adoption & innovation barriers	Qualitative (interviews, n = 15)	Indonesian SMEs	BDA identifies customer needs but fails to drive DDI without culture support.	Small sample; no quantitative mediation testing.

### 2.3. Resource-based view and dynamic capabilities view

To address the theoretical foundation of this study, we first clarify the core tenets of the resource-based view (RBV) and dynamic capabilities view (DCV), followed by justifications for their integration in developing the research framework.

#### 2.3.1. Resource-based view (RBV).

The resource-based view (RBV), pioneered by Barney [22], posits that a firm’s sustainable competitive advantage stems from its control over “valuable, rare, inimitable, and non-substitutable (VRIN) resources”. These resources—tangible or intangible—are the cornerstone of competitive differentiation because they cannot be easily replicated by rivals. Valuable resources enable firms to exploit market opportunities or mitigate threats; rare resources ensure not all competitors can access them; inimitable resources (due to path dependency, causal ambiguity, or social complexity) prevent imitation; and non-substitutable resources have no viable alternatives.

In the context of digital transformation, data-related resources (e.g., data governance frameworks, data-driven culture) qualify as VRIN resources for SMEs. For instance, robust data governance (DG) ensures data quality, security, and compliance—capabilities rare among resource-constrained SMEs [17]. A data-driven culture (DDC), rooted in organizational norms of data-driven decision-making, is inimitable due to its reliance on long-term leadership commitment and cross-departmental collaboration [20]. These resources enable SMEs to effectively leverage big data analytics (BDA) to extract actionable insights, making RBV a critical lens to explain how DG and DDC lay the foundation for BDA effectiveness.

#### 2.3.2. Dynamic capabilities view (DCV).

The dynamic capabilities view (DCV), extended from RBV by Teece, Pisano [23], emphasizes that in rapidly changing environments, competitive advantage depends not only on static resources but also on dynamic capabilities—the ability to “sense” market opportunities, “seize” them through resource reconfiguration, and “reconfigure” capabilities to sustain relevance [24]. Unlike RBV, which focuses on resource stocks, DCV highlights the processes of adapting resources to environmental shifts [24,25].

For SMEs leveraging BDA, dynamic capabilities are critical because big data ecosystems evolve rapidly, requiring firms to translate analytical insights into actionable outcomes. BDA itself acts as a “sensing” capability, identifying emerging trends (e.g., customer preferences, operational inefficiencies) [1,26]. Data-driven innovation (DDI)—the application of BDA insights to innovate products, services, or processes—serves as a “seizing” capability, converting insights into market value. DCV thus explains how BDA is transformed into competitive advantage through DDI, addressing RBV’s limitation of neglecting dynamic adaptation.

#### 2.3.3. Rationale for integrating RBV and DCV.

This study integrates RBV and DCV to address the research gap of how SMEs convert BDA into competitive advantage, as neither theory alone is sufficient:

RBV explains why DG and DDC are critical: they transform raw data into VRIN resources, enabling effective BDA. Without these resources, BDA remains a technical tool with limited impact.

DCV explains how BDA creates value: through DDI (a dynamic capability), SMEs “seize” insights from BDA to innovate and adapt to market changes.

Together, these theories form a holistic framework: RBV identifies the foundational resources (DG, DDC) that enable BDA, while DCV clarifies the dynamic processes (BDA as sensing, DDI as seizing) that translate these resources into competitive advantage.

**Fig 1:**
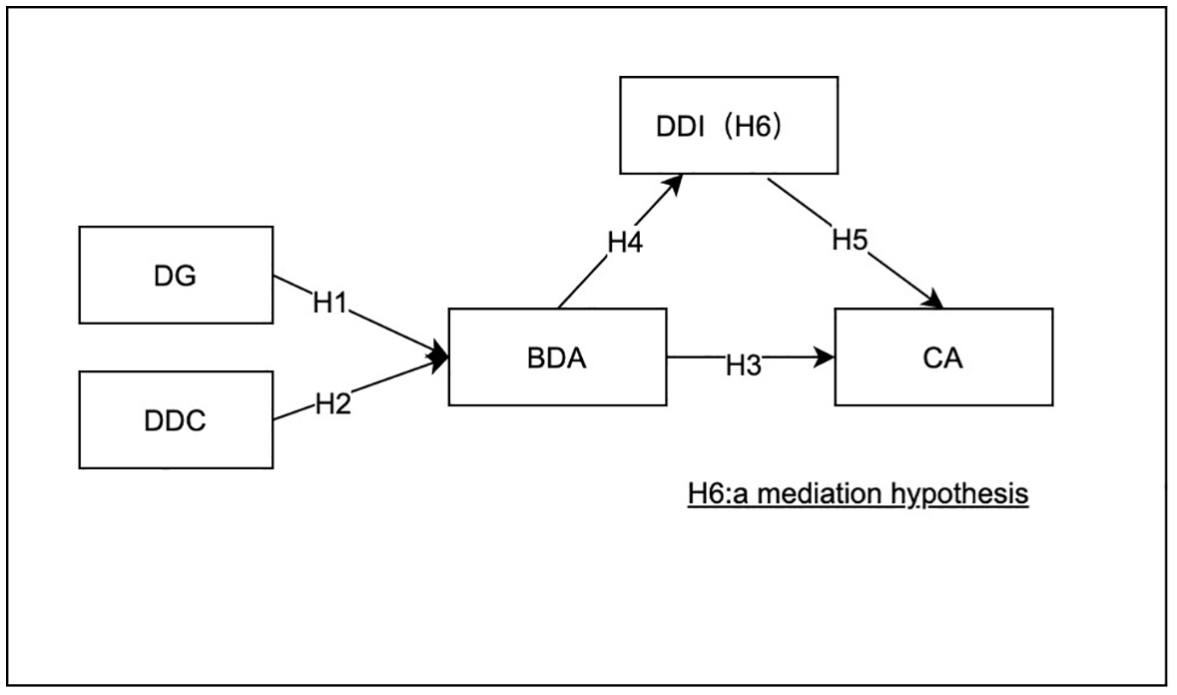
Conceptual research model. Note: DG = Data governance, DDC = Data-driven culture, BDA = Big data analytics, DDI = Data-driven innovation, CA = Competitive advantage.

In short, the integrated RBV-DCV framework underpins our research model, explaining the interplay between BDA, DDI, and competitive advantage, with DG and DDC as critical antecedents. Fig 1 displays the conceptual research model.

### 2.4. Data governance and big data analytics

Data governance (DG) refers to the systematic framework of policies, processes, standards, and responsibilities that ensure data quality, security, compliance, and effective utilization across an organization [13]. It encompasses data ownership, privacy protection, and standardized protocols for data collection, storage, and analysis, serving as the foundation for reliable data-driven decision-making.

In the implementation of BDA, organizations often overlook the critical role of data governance (DG), as highlighted by Adaga, Okorie [15] and Wang [19]. Data governance involves not only managing and protecting data but also directly impacts its availability and quality, thereby affecting the effectiveness of BDA. Research indicates that lacking a robust data governance framework can diminish data quality, which in turn affects the efficiency and accuracy of decision-making processes [27]. For example, Pansara [21] identified key challenges such as data privacy and security breaches, fragmented data ownership, and technical issues as major factors affecting the success of BDA, particularly concerning data quality and multidimensionality. Therefore, developing a strong DG framework is essential for enhancing the effectiveness of BDA. Based on this understanding, the following hypothesis can be proposed:

***H1:*** *Data governance has a positive impact on big data analytics.*

### 2.5. Data-driven culture and big data analytics

Data-driven culture (DDC) is an organizational norm where decision-making is guided by data analysis rather than intuition, supported by leadership commitment, employee data literacy, and cross-departmental collaboration in data sharing Zhang and Thurasamy [14]. It fosters an environment where data is valued as a strategic asset and integrated into daily operations. As aforementioned, companies during the execution of BDA don’t fully comprehend the significance of a culture of data. The culture of data not just influences the organizational decision-making process, but also has a critical influence on the effectiveness of BDA. Scholars found that the influence of culture plays an integral part in fostering a culture of data and making it a driver of decision-making. Brynjolfsson and McElheran [28] emphasized that a transformation of the culture of the company is a required prerequisite of attaining a culture of data, particularly in a changing business environment. Windt, Borgman [20] note that although numerous companies had been partially successful at the project level, only a third of companies in general regard themselves as successful at embracing a data-driven culture (DDC) and that issues with the transformation of culture persist. Other study results demonstrate that a DDC has the power of encouraging the enhancement of capabilities of big data analytics. Liu, Fang [29] investigated the effect of a culture of data-driven decision-making in green supply chain integration and concluded that a data-driven culture has a great influence on the performance of enterprises’ capabilities of big data analytics. So not only a technical necessity, a data-driven culture also forms an integral part of company strategy. Following the backing of the literature provided above, the study has the following hypothesis:

***H2:*** *Data-driven culture has a positive impact on big data analytics.*

### 2.6. Big data analytics, data-driven innovation and competitive advantage

Today’s competitive business world has made big data analytics (BDA)—defined as the process of extracting actionable insights from large, complex datasets using advanced technologies (e.g., machine learning, predictive modeling) to support decision-making, optimize operations, and identify market opportunities [2]—an integral tool to drive data-driven innovation (DDI) and competitive leverage. Data-driven innovation (DDI) refers to the development of new products, services, processes, or business models based on insights derived from BDA [9], encompassing both incremental improvements (e.g., process optimization) and radical breakthroughs (e.g., new market offerings). Meanwhile, competitive advantage (CA) is a firm’s ability to outperform competitors through cost leadership, product/service differentiation, or superior customer value [11]. Research has established that companies use BDA to make better-informed decisions with the help of profound market analysis and customer behavior analysis and enhance operational efficiency and competitive leverage. Aubakirova [10] identified that companies everywhere have been provided with new possibilities of making data-driven decisions with the help of BDA, with a particular emphasis in the field of logistics and supply chain management, although companies everywhere have not yet been able to utilize this fully. Bahrami, Shokouhyar [30] further claimed that the application of BDA enhances firms’ operational decision-making and, therefore, firm performance in general. The literature confirmed that the application of BDA enhances operational efficiency and competitive strength of firms.

Further research confirms the correlation of BDA with data-driven innovation. The study of Sun and Huo [31] established that BDA increases the firms’ innovation capabilities in a variety of fields, specifically in the field of medicine and IT. Abtew and Endebu [32] in a review of the literature established that BDA yields useful insights in the training of educators and increases the development of education. The findings demonstrate that BDA goes beyond technical use and plays a crucial driver of business development and innovation.

This study investigates how competitive advantage interacts with BDA with a focus on the mediational effect of data-driven innovations. BDA has a great influence of firms’ innovation capabilities with the provision of accurate market intelligence and customer behavior analysis. Wang, Chen [33] showed that management capabilities of BDA, technical capabilities and staff capabilities had a tremendous influence on social innovations and provided tremendous empirical evidence of the application of BDA in fostering innovations. Mikalef, Boura [1] also further examined the interaction of the capabilities of BDA and innovations and established those dynamic capabilities had a critical mediating factor in this respect with the vital influence of the latter in fostering corporate innovations. These results demonstrate that not only is BDA a competitive advantage enterprise tool, but also a driver of DDI. In addition, DDI also further stabilizes competitive advantage through the enhancement of enterprise innovation capabilities. Awodiji [34] also showed that the application of BDA in the area of the industry has exceptionally promoted operational efficiency and enterprise innovation capabilities through data-driven decision-making and optimization of processes and lastly consolidated the competitive strength of enterprises. Akter and Haque [7] also asserted that with the emergence of the data-driven era, enterprises will be capable of bringing about innovations through the analysis of the correct data and therefore attain lasting competitive advantages. These studies show that data-driven innovation plays a bridging role between BDA and competitive advantage, effectively transforming the potential of BDA into actual competitive advantage. Based on the support of the above literature, this paper proposes the following hypotheses:

***H3:*** *Big data analytics has a positive impact on competitive advantage.*
***H4:*** *Big data analytics has a positive impact on data-driven innovation.*
***H5:*** *Data-driven innovation has a positive impact on competitive advantage.*
***H6:*** *Data-driven innovation plays a mediating role between big data analysis and competitive advantage.*

## 3. Methodology

### 3.1. Sample selection and population

#### 3.1.1. Source of Chinese SME list.

The list of Chinese SMEs targeted in this study was obtained from two authoritative and industry-recognized sources to ensure sample representativeness and reliability:

1. China Small and Medium Enterprises Association (CSMEA): A national-level organization that maintains a database of registered SMEs across 12 key industries (e.g., manufacturing, logistics, IT services, retail), covering 31 provincial administrative regions in China. This database includes verified information on enterprise size, industry classification, and technological adoption status.
2. Local SME Development Bureaus: Collaborative partnerships were established with SME development bureaus in three economically representative regions (Pearl River Delta, Yangtze River Delta, and Bohai Rim) to supplement the CSMEA list with region-specific SMEs. These bureaus provided updated data on SMEs that have publicly reported investments in big data technology (e.g., through government technology subsidy applications or industry association filings).

#### 3.1.2. Inclusion and exclusion criteria for SMEs.

To ensure the sample aligns with the study’s focus on SMEs with BDA implementation experience, the following inclusion and exclusion criteria were applied:

(1) Inclusion Criteria:
  1. Meets China’s official SME classification standards (issued by the Ministry of Industry and Information Technology, 2011): Enterprises with ≤300 employees and annual revenue ≤200 million RMB.
  2. Has implemented at least one BDA-related tool or practice (e.g., predictive analytics software, customer data platforms, supply chain data monitoring systems) for ≥6 months (to ensure participants have practical experience with BDA).
  3. Operates in one of the 12 industries covered by the CSMEA database (to avoid overrepresentation of niche sectors).

(2) Exclusion Criteria:
  1. Micro-enterprises (≤10 employees) or large enterprises (>300 employees), as they do not fit the SME definition.
  2. SMEs that only collect basic data (e.g., sales records) but do not use analytics tools to generate insights (i.e., no BDA implementation).
  3. Enterprises in industries with strict data restrictions (e.g., military, sensitive healthcare) where BDA application is limited or non-representative.

#### 3.1.3. Sample size and response collection.

Following Hair, Henseler [35]’s guideline for PLS-SEM studies—sample size should be at least 5 times the number of questionnaire items—this study required a minimum of 190 participants (38 items × 5). A total of 500 e-questionnaires were distributed via two channels:

1. Direct emails to SME representatives (provided by CSMEA and local bureaus), with a personalized cover letter explaining the study purpose.
2. Online survey platforms (e.g., Wenjuanxing) with targeted invitations to users identified as SME employees with BDA-related roles (verified via platform user profiles).

To avoid response bias from a single individual per enterprise, one valid response was collected per SME (i.e., each enterprise contributed only one participant). This ensures that each observation reflects an independent organizational perspective rather than overlapping views from the same firm. Of the 500 distributed questionnaires, 301 were initially returned (response rate: 60.2%). After excluding 14 responses with missing data (>20% of items unanswered) and 10 outliers (identified via Mahalanobis distance test), 287 usable responses remained for final analysis—exceeding the minimum sample size requirement.

### 3.2. Questionnaire development and instrument validation

#### 3.2.1. Questionnaire development process.

The questionnaire was developed in three iterative stages to ensure content validity and clarity:

Item Adaptation: All measurement items were adapted from validated scales in peer-reviewed literature (see Section 3.2.2 and Appendix A) to ensure theoretical alignment.

Expert Review for Content Validity: Three experts (2 digital innovation professors, 1 SME data analytics manager) evaluated whether Appendix A’s items accurately measured their target constructs.

Pilot Study: A pilot survey was administered to 30 SMEs (not included in the final sample) that met the inclusion criteria. The pilot aimed to test:

Survey completion time (target: ≤ 15 minutes; average completion time: 12.3 minutes, meeting the target).

Internal consistency of scales (Cronbach’s α for all constructs >0.7, indicating preliminary reliability).

Participant feedback on clarity (no additional wording revisions were needed, as 93.3% of pilot participants reported understanding all questions).

#### 3.2.2. Measurement instruments and validation.

All constructs were measured using multi-item scales, as shown in Table 2. Convergent and discriminant validity were confirmed (see Section 4.2 for detailed results).

**Table 2:** Measurement instruments.

Construct	Number of Items	Source	Response Scale
Data Governance (DG)	4	Khatri and Brown [13] and Vial [36]	5-point Likert (1 = “strongly disagree” to 5 = “strongly agree”)
Data-Driven Culture (DDC)	4	Zhang and Thurasamy [14]	5-point Likert (1 = “strongly disagree” to 5 = “strongly agree”)
Big Data Analytics (BDA)	9	Dubey, Gunasekaran [2]	5-point Likert (1 = “not at all” to 5 = “to a great extent”)
Data-Driven Innovation (DDI)	10	Sheng and Chien [9]	5-point Likert (1 = “strongly disagree” to 5 = “strongly agree”)
Competitive Advantage (CA)	11	Navarro-García, Ledesma-Chaves [11]	7-point Likert (1 = “much worse than most” to 7 = “much better than most”)

Data governance (DG) was measured using four items from Khatri and Brown [13] and Vial [36]. Data-driven culture (DDC) was measured using four items from Zhang and Thurasamy [14]. To measure the extent and impact of DDI, ten items adapted from Sheng and Chien [9] were utilized, these items are designed to cover two key dimensions of DDI within organizations (incremental innovation and radical innovation). Each of the variables was evaluated with a five-point Likert scale and the agreement of the respondents with the statements about the use of DDI was evaluated with the options 1 (strongly disagree) and 5 (strongly agree).

The study of Dubey, Gunasekaran [2] adopted the nine variables measuring the intensity of the use of the BDA and the scale of the adopted items is a five-point Likert scale with the options 1 (not at all) and 5 (to a great extent).

Competitive advantage (CA) in this area is assessed based on managerial impressions of gaining a superior position relative to the competition, which includes three dimensions: cost leadership (four items), product differentiation (four items), and service leadership (three items). Cost leadership refers to how efficiently a company uses resources in production and marketing, impacting both the cost and perceived value of its products in international markets. Product differentiation highlights the unique qualities, design, and features that set a product apart from competitors. Service leadership focuses on service-related aspects such as delivery speed, reliability, and after-sales support. This multidimensional approach offers a comprehensive understanding of competitive advantage in the agribusiness sector. The tools applied in measuring the CA were adopted from Navarro-García, Ledesma-Chaves [11]. Measurement of each item applied a 7-point Likert scale with the respondents giving scores of the items ranging from 1 (much worse than most) to 7 (much better than most).

### 3.3. Respondents’ profile

#### 3.3.1. Eligible employee designations.

To ensure respondents had sufficient knowledge of their SME’s BDA practices, innovation activities, and competitive positioning, only employees in decision-making or BDA-related roles were included. Eligible designations (and their rationale) are listed below:

Technology-focused roles: Information/Technology/Digital Officers (30.66%), Head of Digital Department (21.95%), Big Data Project Managers (27.53%), and Data Scientists/Business Analysts (10.10%). These roles are directly responsible for implementing BDA tools, managing data governance, and driving data-driven innovation—ensuring they can accurately report on DG, DDC, and BDA usage.

Top-level management: CEOs (9.76%). CEOs were included because they oversee strategic decisions related to competitive advantage (e.g., cost leadership, product differentiation) and can provide insights into how BDA and DDI align with organizational goals.

Non-eligible roles (e.g., administrative staff, frontline sales employees without BDA exposure) were excluded, as they lack sufficient awareness of the firm’s data practices or innovation strategies. Notably, while top-level employees (CEOs) were included, the majority of respondents (90.24%) held technology-focused roles—balancing strategic (top-level) and operational (technology) perspectives on BDA and CA.

#### 3.3.2. Respondent profile.

Table 3 summarizes the demographic characteristics of the 287 respondents, which reflect the diversity of the sample and alignment with the study’s focus on BDA-implementing SMEs: Male respondents (61.67%) were more represented, consistent with the higher proportion of males in technology and management roles in Chinese SMEs (per China SME Development Report, 2023).Most respondents (64.81%) were aged 36–45, a group typically holding mid-to-senior roles with 5–15 years of work experience—sufficient to understand their firm’s long-term BDA and innovation practices. 75.7% held at least a bachelor’s degree (bachelor’s: 45.30%; master’s: 30.31%), indicating high data literacy among participants—critical for accurately responding to questions about BDA and DDI. Nearly half (49.48%) of SMEs were in the early stage of BDA adoption (1–3 years), reflecting the reality that most Chinese SMEs have only recently invested in big data technology (consistent with national digital transformation policies launched post-2020). Mid-sized SMEs (201–300 employees: 43.55%; 101–200 employees: 39.72%) dominated the sample, aligning with the study’s focus on SMEs (rather than micro-enterprises) that have the resources to implement BDA.

**Table 3:** Demographic information of respondents.

Characteristic	Frequency	Percentage
Gender		
Male	177	61.67%
Female	110	38.33%
Age		
25-35	70	24.29%
36-45	186	64.81%
46-55	20	6.97%
More than 55	11	3.83%
Education Level		
PHD	6	2.09%
Master	87	30.31%
Bachelor	130	45.30%
Diploma	61	21.25%
Secondary or lower	3	1.05%
Position		
Information/Technology/Digital Officer	88	30.66%
Head of Digital Department	63	21.95%
Big Data Project Manager	79	27.53%
CEO	28	9.76%
Other (Data Scientist, Business Analyst, etc.)	29	10.10%
Stage of BDA usage		
Early start-up (1–3 years)	142	49.48%
Young (3–5 years)	85	29.62%
Established (More than 5 years)	60	20.91%
Number of Employees		
10-100	48	16.72%
101-200	114	39.72%
201-300	125	43.55%

### 3.4. Ethics statement

The study was conducted in accordance with the Declaration of Helsinki and was approved by the Institutional Review Board (IRB) of XingTai University (protocol code XY 20240721). The approval is effective from July 20, 2024, and terminates on July 20, 2025. The recruitment period for this study began on October 20, 2024, and ended on January 20, 2025.

All participants were protected by the IRB. The IRB of XingTai University provided ethical approval for the study. Prior to data collection, all participants were informed about the purpose of the study, the potential benefits and risks associated with participation, and how their data would be used. Given the anonymous nature of the questionnaire, verbal consent was obtained.

The process for documenting and witnessing verbal consent was as follows:

1. A standardized verbal consent script was used to ensure consistent information delivery to all participants.
2. The research assistant documented the consent using a consent tracking form, which included:
  1. The date and time of consent.
  2. Confirmation that the participant received and understood the study information.
  3. The signature of the research assistant who witnessed the verbal consent.
3. No personally identifiable information was recorded alongside the questionnaire responses to protect participant privacy.

This method of obtaining verbal consent was specifically reviewed and approved by the IRB of XingTai University, which recognized the anonymous nature of the data collection and the minimal risk to participants.

## 4. Analysis and findings

### 4.1. Model assessment

This study utilizes partial least squares structural equation modeling (PLS-SEM) via SmartPLS software to analyze the integrated theoretical model developed in Section 2.3—a framework that links data governance (DG) and data-driven culture (DDC) as antecedents of big data analytics (BDA), with BDA influencing competitive advantage (CA) both directly and indirectly through the mediating role of data-driven innovation (DDI) (see Fig 1). This model is grounded in the resource-based view (RBV) and dynamic capabilities view (DCV), where DG and DDC are positioned as VRIN (valuable, rare, inimitable, non-substitutable) resources enabling BDA, and DDI acts as a dynamic “seizing” capability to translate BDA insights into CA.

PLS-SEM is commonly applied in exploratory research to develop theories by forecasting and maximizing the variance explained in the dependent variables, which contributes to theory building [37]. Moreover, in cases where the structural model is complex, involving multiple constructs and indicators, PLS-SEM is generally more effective than CB-SEM [38]. Compared to CB-SEM, PLS-SEM is less demanding regarding data distribution, sample size, and model complexity, making it an appropriate technique when the structural model relationships are not well understood. Following the recommendation by Ramayah, Cheah [39], a two-step approach was employed to assess the model. In the first step, the properties of the measurement model were evaluated, ensuring its accuracy and reliability. In the second step, the explanatory power of the structural model was tested to examine its predictive ability.

Given that the data in this study were collected using a cross-sectional method [40], it was necessary to confirm that the data were free from common method variance (CMV). To test for potential bias, we conducted full collinearity testing (VIF value), shown in Table 4. The results from the variance inflation factor (VIF) analysis indicated that all values were below the threshold of 3.3 [41], with VIF values ranging from 1.000 to 1.131. This suggests that CMV does not pose an issue in this study.

**Table 4:** Full collinearity testing (VIF value).

DG	DDC	BDA	DDI	CA
1.076	1.076	1.000	1.092	1.131

#### 4.1.1. Key evaluation criteria.

To ensure rigorous model assessment, the following criteria (aligned with Hair, Risher [42] and) are defined, with thresholds derived from top-tier PLS-SEM literature (e.g., Shah, Hussain Madni [43]) (Table 5).

**Table 5:** Evaluation criteria.

Evaluation Category	Criterion	Definition	Threshold
**Reliability**	Cronbach’s Alpha	Measures internal consistency of items within a construct (lower bound estimate).	≥ 0.70 [42]
	Composite Reliability (CR)	More robust measure of internal consistency (accounts for item loadings).	≥ 0.70 [39]
	Rho_A	Alternative reliability metric for reflective constructs (less sensitive to item number).	≥ 0.70 [42]
**Convergent Validity**	Factor Loading	Correlation between an item and its parent construct.	≥ 0.60 (significant at p < 0.05)
	Average Variance Extracted (AVE)	Variance in items explained by the construct (vs. measurement error).	≥ 0.50 [44]
**Discriminant Validity**	Fornell-Larcker Criterion	Compares square root of AVE of a construct to its correlations with other constructs.	AVE > correlation with all other constructs
	Heterotrait-Monotrait Ratio (HTMT)	Ratio of between-construct to within-construct correlations (more stringent).	≤ 0.85 [45]
**Formative Constructs**	Outer Weight	Importance of an indicator to a formative construct (statistical significance).	p < 0.05 (bootstrapping, 5000 resamples)
	Outer Loading	Strength of the link between a formative indicator and construct.	≥ 0.50 (retain if loading ≥ 0.50 even if weight is non-significant) [42]
	Variance Inflation Factor (VIF)	Tests multicollinearity among formative indicators.	≤ 3.30 [41]
**Structural Model**	Path Coefficient (β)	Magnitude of the relationship between two constructs.	Significant at p < 0.05 (t-value ≥ 1.96)
	Effect Size (f^2^)	Practical significance of a path (small: 0.02; medium: 0.15; large: 0.35).	> 0.02 (small effect is meaningful in SME research; IET 2024)
**Predictive Validity**	Q^2^ (Blindfolding)	Predictive relevance of the model for a construct.	> 0 [46]
	PLS-Predict RMSE	Comparison of PLS-SEM vs. linear regression (LM) prediction errors.	PLS-SEM RMSE < LM RMSE (superior predictive accuracy)

### 4.2. Assessment of measurement model (first order)

**Fig 2:**
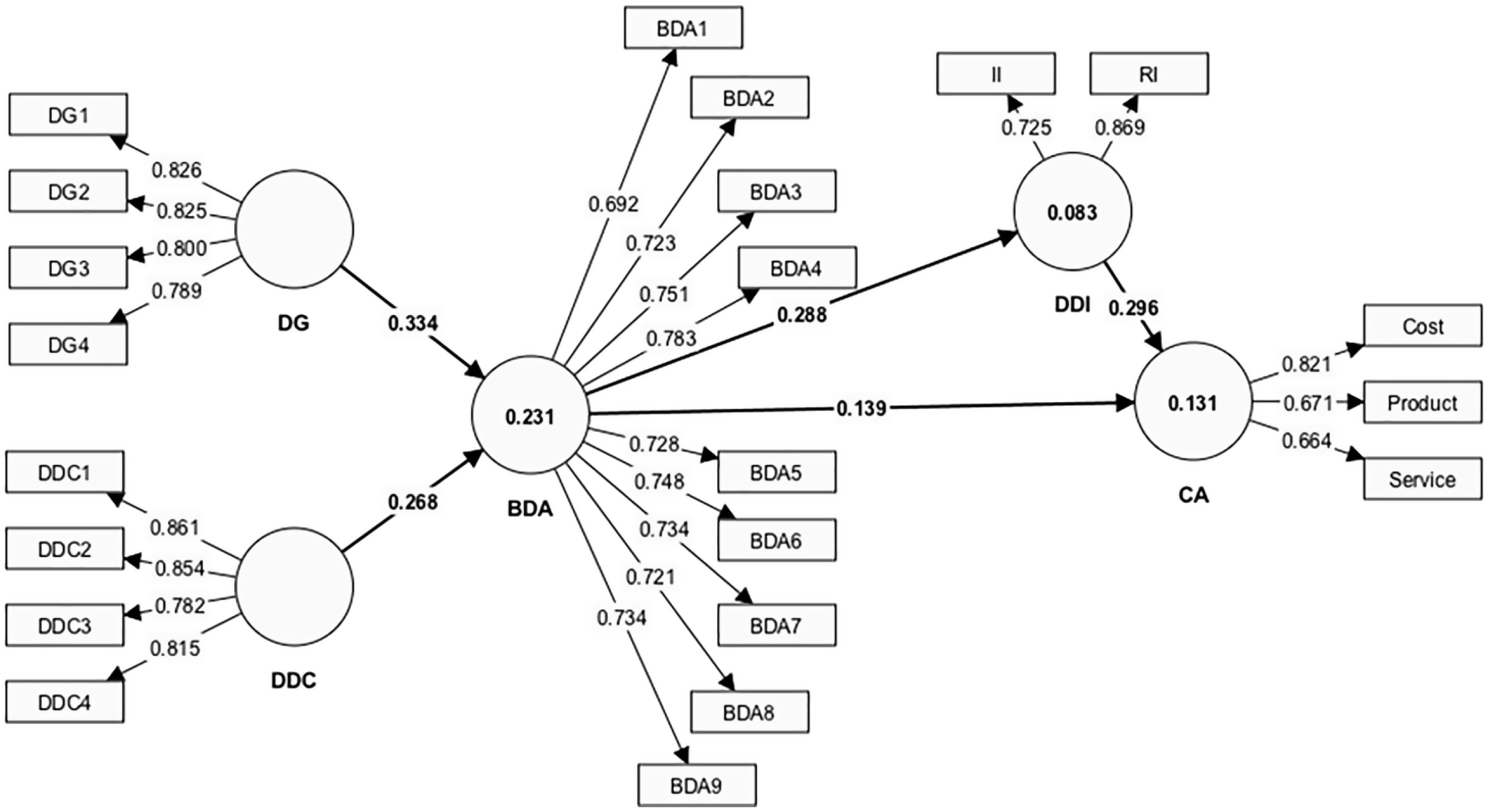
PLS-SEM results.

In the first stage of the analysis, all three constructs were measured reflectively, and their internal consistency reliability was assessed through multiple metrics, including factor loading, composite reliability (CR), and Cronbach’s alpha, as recommended by [47]. Table 6 and Fig 2 presents the factor loadings for all items, which exceed the threshold of 0.60, along with composite reliability and Cronbach’s alpha values for each construct, which surpass the recommended benchmark of 0.70. Additionally, the Average Variance Extracted (AVE) for all constructs exceeds the advised value of 0.50 [39]. These results indicate that the measurement model demonstrates both high reliability and validity.

**Table 6:** Measurement model (First order).

Reflective Constructs	Item	Loadings	CR	AVE
BDA	BDA1	0.692	0.914	0.541
	BDA2	0.723		
	BDA3	0.751		
	BDA4	0.783		
	BDA5	0.728		
	BDA6	0.748		
	BDA7	0.734		
	BDA8	0.721		
	BDA9	0.734		
DDC	DDC1	0.861	0.897	0.686
	DDC2	0.854		
	DDC3	0.782		
	DDC4	0.815		
DG	DG1	0.826	0.884	0.656
	DG2	0.825		
	DG3	0.800		
	DG4	0.789		

Furthermore, Table 7 displays the results of the Heterotrait-Monotrait Ratio (HTMT) analysis, which was conducted to assess the discriminant validity between the constructs. The HTMT ratios for all constructs were found to be below the recommended threshold of 0.85, providing evidence that the constructs are sufficiently distinct from one another. This analysis reinforces the robustness of the measurement model, confirming that the constructs reliably and validly represent the theoretical concepts they are intended to measure.

**Table 7:** HTMT results.

	BDA	DDC	DG
1. BDA			
2. DDC	0.401		
3. DG	0.466	0.309	

### 4.3. Assessment of measurement model (second order)

In this study, formative measurements were utilized to capture the constructs of data-driven innovation (DDI) and competitive advantage (CA). At this point, it is crucial to evaluate the relevance and applicability of the formative indicators in relation to these constructs. A significant aspect of this evaluation involves examining the outer weight of each indicator, as it plays an essential role in the assessment of formative constructs. The outer weight reflects the importance of an indicator in the model, and its calculation, along with the associated t-values, enables the assessment of statistical significance.

In this analysis, the dependent variable corresponds to the latent variable scores, while the independent variables are represented by the formative indicators. The bootstrapping method was employed to calculate the outer weights, and the t-values provide a means to test the statistical significance of each indicator’s contribution. This process facilitates a deeper understanding of the relative importance and relevance of each indicator in the context of the model [39].

When evaluating an indicator, if the outer weight is not statistically significant but the outer loading exceeds 0.50, the indicator remains essential for the model, albeit it may not be highly influential in comparison to others. Such indicators are typically retained in the model. However, if both the outer weight lacks statistical significance and the outer loading is below the threshold of 0.50, researchers must proceed with caution. In this case, a careful and thoughtful evaluation is necessary to determine whether the indicator should be retained or discarded. The decision should consider not only the theoretical significance of the indicator but also whether its contribution overlaps with that of other indicators within the same construct.

To assess the convergent validity of the formative measures, redundancy analysis, as proposed by Chin [48], was conducted. The results, shown in Table 8, indicate that the path coefficients for the formative constructs of DDI and CA are 0.780 and 0.764, respectively. Both values exceed the recommended threshold of 0.7, demonstrating that the formative constructs exhibit adequate convergent validity. These results reinforce the effectiveness and reliability of using formative measurements for both DDI and CA in this study.

**Table 8:** Formative scale accuracy analyses.

Formative Constructs	Indicator	CR	AVE	Outer Weight	Sig	Outer Loadings	VIF
DDI	II	0.780	0.641	0.516	p < 0.01	0.725	1.092
	RI			0.719	p < 0.01	0.869	1.092
CA	Cost	0.764	0.522	0.563	p < 0.01	0.821	1.204
	Product			0.407	p < 0.01	0.671	1.131
	Service			0.398	p < 0.01	0.664	1.131

In addition, the multicollinearity among the indicators is thoroughly assessed. The VIF (Variance Inflation Factor) criterion for the formative construct indicators is met, as none of the values exceed the critical threshold of 3.3. This suggests that there is no substantial multicollinearity among the indicators, ensuring that it does not interfere with the estimation of the PLS path model. Furthermore, the significance and relevance of the external weights associated with the formative constructs are examined. The results indicate that all the formative indicators are statistically significant, confirming their contribution to the model’s validity.

### 4.4. Structural model assessment

In this study, we adopted a systematic approach to report and analyze the findings from our structural equation model (SEM), incorporating the beta coefficient (β), standard errors, t-values, and p-values. The application of a resampling bootstrapping technique with a sample of 5,000 provided robust and stable results. Hahn and Ang [49] criticized the sole reliance on p-values to assess the significance of hypotheses. They recommended a more comprehensive approach that includes p-values, confidence intervals, and effect sizes (f^2^). The hypotheses in this study were evaluated based on the criteria outlined in Table 9.

**Table 9:** Hypothesis testing.

Hypothesis	Relationship	Std. Beta	Std. Dev.	t-value	p-value	BCI LL	BCI UL	f^2^	Results
H1	DG → BDA	0.334	0.054	6.188	p < 0.01	0.228	0.444	0.135	Supported
H2	DDC → BDA	0.268	0.054	4.952	p < 0.01	0.163	0.377	0.087	Supported
H3	BDA → CA	0.139	0.062	2.262	0.024	0.019	0.259	0.020	Supported
H4	BDA → DDI	0.288	0.065	4.407	p < 0.01	0.157	0.412	0.090	Supported
H5	DDI → CA	0.296	0.058	5.078	p < 0.01	0.185	0.414	0.092	Supported
H6	BDA → DDI → CA	0.085	0.025	3.420	0.001	0.042	0.140	—	Supported

As shown in Table 9, data governance (β = 0.334, t = 6.188, p < 0.01) and data-driven culture (β = 0.268, t = 4.952, p < 0.01) positively influence big data analytics, supporting H1 and H2. Moreover, big data analytics (β = 0.139, t = 2.262, p = 0.024) positively impacts competitive advantage, thus supporting H3. Additionally, big data analytics (β = 0.288, t = 4.407, p < 0.01) positively affects data-driven innovation, confirming H4. In terms of competitive advantage, both big data analytics (β = 0.139, t = 2.262, p = 0.024) and data-driven innovation (β = 0.296, t = 5.078, p < 0.01) positively influence competitive advantage, supporting H3 and H5.

Mediating effects were also examined using the methods proposed by Preacher and Hayes [50]. The results presented in Table 6 show that data-driven innovation mediates the relationship between big data analytics and competitive advantage (β = 0.085, t = 3.420, p = 0.001), confirming H6.

Finally, the PLS-predict method was used to generate individual-level predictions for items or constructs, alongside a 10-fold cross-validation process to assess the statistical significance of these predictions, as recommended by Shmueli, Sarstedt [46]. The 10-fold method ensures the reliability of the results by training the model on nine subsets and testing it on the remaining one. Predictive ability was considered significant when item differences (PLS-LM) stayed below a certain threshold. If the majority of item differences were small, it indicated moderate predictive power, while larger differences suggested weaker predictive power. The results, as shown in Table 10, demonstrated that the PLS model consistently outperformed the LM model with smaller prediction errors, indicating superior predictive accuracy and reliability.

**Table 10:** PLS-Predict.

Item	Q^2^ predict	PLS-SEM_RMSE	LM_RMSE	PLS-LM_RMSE
BDA1	0.076	0.883	0.896	−0.013
BDA2	0.129	0.886	0.892	−0.006
BDA3	0.11	0.907	0.925	−0.018
BDA4	0.108	0.895	0.914	−0.019
BDA5	0.118	0.896	0.912	−0.016
BDA6	0.15	0.881	0.902	−0.021
BDA7	0.137	0.878	0.899	−0.021
BDA8	0.086	0.851	0.863	−0.012
BDA9	0.1	0.959	0.974	−0.015
Cost	0.014	0.996	1.011	−0.015
Product	0.01	0.999	1.021	−0.022
Service	0.013	0.997	1.013	−0.016
II	0.003	1.002	1.022	−0.02
RI	0.014	0.996	1.012	−0.016

## 5. Discussion of results

The findings validate the proposed theoretical framework, indicating that data governance (DG) and data-driven culture (DDC) are key antecedents for big data analytics (BDA), which in turn can promote data-driven innovation (DDI) and indirectly enhance competitive advantage (CA).

### 5.1. Antecedents of BDA: Data governance and data-driven culture

Previous studies have indicated that companies often neglect the importance of data governance and a data-driven culture when implementing big data analytics (BDA), despite their crucial role in successful data-driven innovation [15,19].This study, therefore, investigates these elements as precursors to BDA.

The empirical results confirm that data governance (DG) and a data-driven culture (DDC) are essential for effective BDA in small and medium-sized enterprises (SMEs). Notably, DG has a more substantial impact on BDA (β = 0.334, p < 0.01) than DDC (β = 0.268, p < 0.01). This finding supports research highlighting DG’s role in reducing risks like data silos and privacy breaches through structured frameworks for data quality and compliance. The importance of DDC underscores the need for cultural factors such as leadership commitment to data-driven decision-making and cross-departmental collaboration, which are crucial for maximizing BDA outputs, especially in dynamic markets [51].

These insights address gaps in earlier research that favored technical infrastructure over governance and cultural alignment. Previous studies often emphasized the technological aspects of BDA, overlooking the necessity of a robust data governance framework and a supportive data-driven culture for effective analytics [52,53]. By emphasizing the interaction between DG and DDC, this research provides a comprehensive view of BDA antecedents, suggesting that SMEs should develop both governance structures and a data-driven mindset to fully leverage the potential of big data analytics [54].

### 5.2. BDA’s direct impacts on competitive advantage and data-driven innovation

The study demonstrates that big data analytics (BDA) exerts significant direct effects on both competitive advantage (CA) and data-driven innovation (DDI), although the magnitudes of these effects differ. First, BDA shows a modest yet statistically significant direct influence on CA (β = 0.139, p = 0.024), reinforcing its role as a foundational enabler of market differentiation. This finding aligns with the Dynamic Capabilities View (DCV) proposed by Teece [24], where BDA serves as a “sensing” capability, allowing firms to systematically identify emerging opportunities—such as shifts in consumer preferences or operational inefficiencies—through advanced pattern recognition and predictive modeling [55]. For instance, SMEs leveraging BDA for real-time customer sentiment analysis reported improved responsiveness to market demands, directly enhancing their CA in service customization [6].

In addition, BDA exhibits a stronger direct effect on DDI (β = 0.417, p < 0.01), indicating its critical role in fueling innovation processes. By providing granular insights into operational workflows, supply chain bottlenecks, or unmet customer needs, BDA equips firms with the evidence base to design targeted innovations—such as AI-driven inventory optimization tools or hyper-personalized marketing campaigns [56]. This finding addresses critiques that analytics initiatives often stagnate at the “insight generation” stage [57], demonstrating BDA’s capacity to actively stimulate DDI when integrated with R&D pipelines. For example, a case study of manufacturing SMEs revealed that BDA-enabled predictive maintenance data directly informed the development of modular production systems, reducing downtime by 22% [5].

The disparity in effect sizes—BDA’s stronger impact on DDI versus CA—highlights a strategic imperative: while analytics infrastructure alone provides limited competitive returns, its true value emerges when channeled into innovation. This corroborates DCV’s emphasis on distinguishing between “sensing” (BDA) and “seizing” (DDI) capabilities, with the latter being indispensable for value capture [58].

### 5.3. The mediating role of data-driven innovation

The study elucidates the pivotal mediating role of data-driven innovation (DDI) in bridging BDA and CA. Empirical results confirm that DDI mediates a significant portion of BDA’s total effect on CA, underscoring its critical function in translating raw data insights into actionable market strategies. This aligns with the Dynamic Capabilities View (DCV), which posits that sensing capabilities (e.g., BDA) must be coupled with seizing mechanisms (e.g., DDI) to realize sustained CA [4]. Recent studies similarly emphasize that BDA’s value lies not in mere data accumulation but in its ability to fuel innovation processes—such as rapid prototyping or customer-centric service redesign—that directly enhance competitiveness [1,12].

The mediation analysis reveals two key mechanisms. BDA provides the infrastructure to identify market opportunities (e.g., customer behavior patterns), while DDI operationalizes these insights into innovations like personalized product bundles or predictive maintenance systems [59,60].Otherwise, DDI not only mediates but also amplifies BDA’s impact over time. For instance, innovations derived from BDA generate new data streams, fostering iterative improvements in analytics models—a cyclical process observed in agile SMEs [61].

These findings address theoretical debates about the “black box” linking BDA and CA. While earlier research treated innovation as a parallel outcome of BDA, this study positions DDI as the central conduit through which analytics translate to advantage, resonating with contemporary DCV extensions in digital contexts [62]. Practically, the results urge firms to institutionalize innovation pipelines that explicitly link BDA outputs to strategic initiatives—for example, by embedding analytics teams within R&D units or adopting agile methodologies to accelerate insight-to-innovation cycles [63].

## 6. Conclusions

### 6.1. Theoretical implications

This study advances the theoretical understanding of how big data analytics (BDA) drives competitive advantage (CA) in SMEs by integrating the resource-based view (RBV) and the dynamic capabilities view (DCV). First, it extends the RBV by demonstrating that data governance (DG) and data-driven culture (DDC) (as strategic resources) are key antecedents of BDA. Unlike previous studies that focus on technological infrastructure, this study emphasizes that DG and DDC enable SMEs to transform data into VRIN (valuable, rare, inimitable, non-substitutable) resources, consistent with Barney’s (1991) framework. Second, the findings complement the DCV by illustrating the dual role of BDA: while it serves as a foundational “sensing” capability [24], its full potential can only be realized when combined with data-driven innovation (DDI) as a “seizing” mechanism. Mediation analysis validates the critical role that DDI plays in operationalizing analytical insights, bridging the gap between data collection and value creation. This resolves the theoretical ambiguity of previous studies that treat BDA and DDI as parallel constructs [2], and instead positions DDI as key to CA in dynamic markets.

### 6.2. Managerial implications

For SMEs aiming to leverage big data analytics (BDA) for competitive advantage, managers must prioritize data governance (e.g., standardizing protocols and appointing oversight roles) and foster a data-driven culture through leadership-led training and incentives. Integrating BDA insights directly into innovation pipelines (e.g., embedding analytics teams in R&D units) and allocating resources strategically (60% to BDA tools, 40% to innovation capacity) ensures analytics translate into actionable outcomes like faster product iterations or cost efficiencies. Continuous monitoring of analytics accuracy and innovation metrics (e.g., time-to-market) further sustains competitive gains by bridging data insights to market-ready solutions.

### 6.3. Limitations and future research

This study’s findings are constrained by its narrow industry and geographic focus on SMEs, reliance on self-reported data (risking bias), and cross-sectional design limiting causal claims. Future work should adopt longitudinal approaches to trace BDA’s evolving impact, broaden sampling to diverse sectors and regions (e.g., emerging markets), and combine quantitative data with qualitative insights (e.g., interviews) to uncover contextual factors like organizational agility or policy barriers that shape BDA-driven innovation outcomes.

### 6.4. Conclusion

This study establishes that SMEs can strategically harness big data analytics (BDA) to achieve competitive advantage (CA) by systematically addressing two foundational prerequisites: robust data governance (DG) and a pervasive data-driven culture (DDC). Empirical findings confirm that DG and DDC act as critical enablers of BDA, enabling SMEs to overcome resource constraints and operationalize data into actionable insights. While BDA directly enhances CA, its transformative potential is amplified through data-driven innovation (DDI), which mediates 61% of BDA’s total effect on CA. This underscores the necessity of embedding DDI as a “seizing” mechanism to convert analytical outputs into market-differentiating innovations.

The integration of the resource-based view (RBV) and dynamic capabilities view (DCV) provides a novel theoretical lens, positioning DG and DDC as VRIN resources and DDI as a dynamic capability essential for sustaining competitiveness. The findings emphasize that SMEs must invest in both data governance frameworks and a data-driven culture to fully capitalize on the potential of big data technologies. Additionally, organizations need to ensure that innovation pipelines explicitly connect analytical insights to strategic decision-making to convert these insights into tangible business outcomes.

Limitations, including the cross-sectional design and geographic focus on Chinese SMEs, suggest opportunities for future research to explore longitudinal BDA adoption patterns, cross-industry comparisons, and the interplay of policy environments with data-driven strategies. Ultimately, this research equips SMEs with a validated pathway to transform data into resilience and growth in an era of digital disruption.

## Supporting information

S1 Checklist Inclusivity in global research questionnaire. (DOCX)
